# Mindfulness and skills-based eHealth intervention to reduce distress in cancer-affected patients in the Reduct trial: Intervention protocol of the make it training optimized

**DOI:** 10.3389/fpsyt.2022.1037158

**Published:** 2022-10-28

**Authors:** Jana Heinen, Alexander Bäuerle, Caterina Schug, Julia Barbara Krakowczyk, Sven Erik Strunk, Alexandra Wieser, Jil Beckord, Christoph Jansen, Sebastian Dries, Michael Pantförder, Yesim Erim, Stephan Zipfel, Anja Mehnert-Theuerkauf, Jörg Wiltink, Alexander Wünsch, Andreas Dinkel, Andreas Stengel, Johannes Kruse, Martin Teufel, Johanna Graf

**Affiliations:** ^1^Department of Psychosomatic Medicine and Psychotherapy, University Hospital Tübingen, Eberhard Karls University, Tübingen, Germany; ^2^Comprehensive Cancer Center (CCC-TS), University Hospital Tübingen, Tübingen, Germany; ^3^Clinic for Psychosomatic Medicine and Psychotherapy, LVR-University Hospital Essen, University of Duisburg-Essen, Essen, Germany; ^4^Center for Translational Neuro- and Behavioral Sciences (C-TNBS), University of Duisburg-Essen, Essen, Germany; ^5^Comprehensive Cancer Center, University Hospital Essen, Essen, Germany; ^6^Department of Psychosomatic Medicine and Psychotherapy, University Hospital Erlangen, Friedrich-Alexander-University Erlangen-Nürnberg, Erlangen, Germany; ^7^Fraunhofer Institute for Software and Systems Engineering (ISST), Dortmund, Germany; ^8^Department of Medical Psychology and Medical Sociology, University Medical Center Leipzig, Leipzig, Germany; ^9^Department of Psychosomatic Medicine and Psychotherapy, University Medical Center of the Johannes Gutenberg-University Mainz, Mainz, Germany; ^10^Clinic for Psychosomatic Medicine and Psychotherapy, Freiburg Medical Center, Albert-Ludwigs-Universität Freiburg, Freiburg, Germany; ^11^Department of Medical Oncology, Inselspital, Bern University Hospital, University of Bern, Bern, Switzerland; ^12^Department of Psychosomatic Medicine and Psychotherapy, School of Medicine, Klinikum rechts der Isar, Technical University of Munich, Munich, Germany; ^13^Charité Center for Internal Medicine and Dermatology, Department for Psychosomatic Medicine, Charité-Universitätsmedizin Berlin, Corporate Member of Freie Universität Berlin, Humboldt-Universität zu Berlin and Berlin Institute of Health, Berlin, Germany; ^14^Department of Psychotherapy and Psychosomatics, Justus Liebig University Giessen, Giessen, Germany; ^15^Department of Psychotherapy and Psychosomatics, Philipps University Marburg, Marburg, Germany

**Keywords:** intervention protocol, psycho-oncology, cancer, web-based intervention, e-mental health, MBSR, skills training

## Abstract

**Introduction:**

Cancer-affected patients experience high distress due to various burdens. One way to expand psycho-oncological support is through digital interventions. This protocol describes the development and structure of a web-based psycho-oncological intervention, the Make It Training optimized. This intervention is currently evaluated in the Reduct trial, a multicenter randomized controlled trial.

**Methods:**

The Make It Training optimized was developed in six steps: A patient need and demand assessment, development and acceptability analysis of a prototype, the formation of a patient advisory council, the revision of the training, implementation into a web app, and the development of a motivation and evaluation plan.

**Results:**

Through a process of establishing cancer-affected patients’ needs, prototype testing, and patient involvement, the Make It Training optimized was developed by a multidisciplinary team and implemented in a web app. It consists of 16 interactive self-guided modules which can be completed within 16 weeks.

**Discussion:**

Intervention protocols can increase transparency and increase the likelihood of developing effective web-based interventions. This protocol describes the process and results of developing a patient-oriented intervention. Future research should focus on the further personalization of web-based psycho-oncological interventions and the potential benefits of combining multiple psychotherapeutic approaches.

## Introduction

A cancer diagnosis is often associated with multiple physical and psychosocial problems and significant distress, which can persist for years after completion of treatment (1, 2). At the same time, adherence to cancer treatment can decrease due to severe distress (3). These findings highlight that oncological treatment needs to be complemented by efficient psycho-oncological support.

Nevertheless, some burdened patients do not receive psycho-oncological support due to various barriers, including not being screened for distress, limited resources and low accessibility, especially in rural areas (4–6). At the same time, some patients do not utilize the support available despite high psychological distress (4, 7). Potential reasons for not seeking psychological support previously identified are perceived stigma, but also a preference for self-help or the perception the experienced distress is not severe enough in spite of reporting high distress (4, 8).

One way to complement the existing psycho-oncological support for cancer patients is the adoption of digital approaches. Many patients report utilizing the internet to access health information and emotional support (9–11). Digital interventions do not only have the advantage of cost-efficiency and high accessibility and could, therefore, particularly benefit those currently underserved (12, 13). They also allow for more flexible use compared to face-to-face interventions, making integration with frequent oncological treatment and appointments easier. In addition, they could provide support for those who would not seek face-to-face psycho-oncological help (14).

Although more qualitatively sound research is needed (15, 16), previous studies suggest that digital psycho-oncological interventions are feasible and effective (16, 17). More specifically, they have the potential to reduce distress (18–21) anxiety, and depression (19, 22–24). Also, they can improve cancer patients’ overall quality of life (18, 20) and have the potential to reduce physical symptoms of cancer, including fatigue and pain (23, 24). To be effective, digital psycho-oncological interventions must be adapted to cancer patients’ needs (12).

According to the clinical guidelines for psycho-oncology, psychological interventions should be patient-oriented and deliver psychoeducation, relaxation techniques, and evidence-based skills training to help patients deal with cancer-related challenges (25). Most existing psycho-oncological interventions are based on Cognitive Behavioral Therapy (CBT). By applying different cognitive and behavioral techniques to challenge dysfunctional cognitions associated with anxiety and depression, CBT can help reduce cancer patients’ anxiety and depression while helping them better adjust to the illness (26). A meta-analysis by Tatrow and Montgomery (27) suggests that CBT is related to reductions in distress and pain in cancer patients. Various studies have found these benefits to apply to web-delivered CBT for cancer patients (18, 28, 29). However, it must be noted that some cognitions that arise due to the cancer are not dysfunctional but constitute a normal reaction to a potentially life-threatening condition. In the last two decades, the so-called third wave of CBT has brought about a shift toward a more holistic approach to psychotherapy that integrates classical CBT techniques with a novel, trans-diagnostic approach tailored to patients’ specific needs (30, 31) Mindfulness-Based Stress Reduction (MBSR) and Acceptance and Commitment Therapy (ACT) are two psychotherapeutic approaches linked to favorable outcomes in cancer patients. MBSR encourages purposeful, non-judgmental attention to the present moment. This purposeful attention is assumed to lead to a shift of perspective and foster non-reactivity to emotions which is associated with less rumination and distress (32, 33). It has been shown that mindfulness helps cancer patients cope with and regulate challenging emotions related to their illness (34–36). Furthermore, many studies suggest that MBSR reduces stress and anxiety in cancer patients while improving their quality of life (37, 38).

Moreover, research indicates that these beneficial effects of mindfulness extend to web-delivered MBSR (39). ACT, on the other hand, integrates mindful awareness with other elements to promote psychological flexibility, i.e., awareness and acceptance of arising thoughts and emotions. At the same time, it involves clarifying core values and committed action in line with them (31, 40). A growing number of studies suggest that ACT improves the quality of life and wellbeing of cancer patients (41, 42). So far, few web-based psycho-oncological interventions have been integrated with ACT approaches (43, 44).

With an interdisciplinary team of psycho-oncologists, psychotherapists, medical specialists for psychosomatic medicine, graphic designers, and health informatics scientists, and with the involvement of cancer patient representatives, we have developed the Make It Training optimized: A web-based psycho-oncological intervention based on a combination of CBT techniques as well as ACT and MBSR. The self-guided intervention is aimed at equipping cancer patients with skills to manage and deal with cancer-related challenges. It is a thereby assumed to reduce distress. More information about the ongoing effectiveness trial is available in the published study protocol (45). In this protocol, we will report the development and structure of the intervention. To this end, the template for intervention description and replication (TIDieR) checklist [(46); see Supplementary Table 1] was used.

## Methods

**FIGURE 1:**
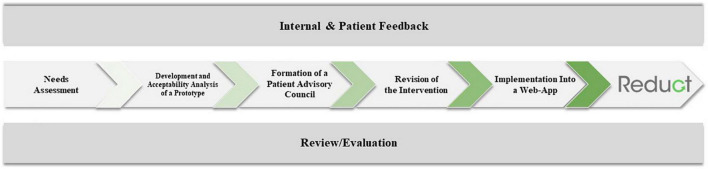
The development process of the make it training. *Note*. Reduct Refers to the ongoing evaluation of the Make It Training. Please consult the published study protocol for further information (45).

The Make It development involved six steps (Figure 1): (1) A needs assessment, (2) an acceptance analysis of the first version of the intervention, (3) the establishment of a patient advisory council, (4) the selection of evidence-based methods and exercises, (5) the development and implementation of the current version of the intervention, and (6) the development of a motivational plan to foster adherence as well as an evaluation plan.

In step 1, a comprehensive online survey was conducted to assess the needs and demands of cancer patients concerning psycho-oncological eHealth applications. The aim was to improve the acceptance and effectiveness of digital psycho-oncological interventions.

In step 2, a web-based self-guided intervention, the Make It Training, was developed based on step the needs assessment of step 1. The intervention’s benefits, design, and layout were evaluated in a first acceptance analysis and a longitudinal observational study. The aim was to see whether this first version of the Make It Training was a satisfactory psycho-oncological eHealth intervention and to identify potential future points of revision.

In step 3, a council of four female patients and one male patient was formed. They are members of cancer support groups and were contacted by the psycho-oncological units of the university hospitals of Essen, Tübingen, and Erlangen. Two members were nominated by a German umbrella organization for cancer self-help. The primary role of the patient council was to advise the project team concerning further planning and optimization of the intervention and related studies. The patient council was consulted regularly during the intervention development regarding content, usability, and communication with the target group.

In step 4, the content of the intervention was revised based on the previous steps. It was ensured that each module was structured consistently so that the use is intuitive and the design is user-friendly. An intervention structure was developed based on the selected methods and practical exercises. Previous evidence-based digital interventions were used as starting point.

In step 5, the programming of the web app was planned together with the Department of Digitization and Healthcare of the Fraunhofer Institute for Software and Systems Engineering (ISST). The ISST is part of the Fraunhofer-Gesellschaft, an international research and technology organization. The programming and graphic design aspects were discussed at regular meetings. Furthermore, the intervention was continuously tested by different users on different operating systems to evaluate the intervention program’s usability and identify points for improvement.

In Step 6, a motivation and an evaluation plan were developed to enhance user adherence and assess the intervention’s relevant effectiveness parameters. The evaluation plan is based on validated assessment instruments to evaluate different parameters such as efficacy, satisfaction, usability, and predictors of usage of the intervention. In addition, self-generated items were developed to measure the benefits of the intervention in detail. As this paper aims to describe the development of the digital intervention, the evaluation plan will only be addressed briefly here. More information regarding the ongoing evaluation can be found on the German Clinical Trial Register (DRKS; DRKS-ID: DRKS00025213) or in the published study protocol (45).

## Results

### Step 1—Needs and demands assessment

The pre-study by Ringwald et al. (47) aimed to establish relevant content topics that psycho-oncological e-health interventions should address. Seven hundred sixteen patients were included in the study. The content topics with the highest preference were anxiety, ability to cope, quality of life, depressive feelings, and adjustment to a new life situation. Spirituality, sense-making, and dealing with children were also relevant for eHealth interventions in this sample. In addition, eHealth applications (including web-based applications, websites, blogs, information e-mails, and consultation hotlines) were considered suitable for conveying these content topics. The patients’ subjective psychological burden regarding distress, quality of life, and need for psychosocial support did not influence the preference rates for psycho-oncological contents and eHealth applications.

### Step 2—Development and acceptance testing of the first version of the digital intervention

The second step was to develop a first version of the web-based program “Make It Training” (Mindfulness and skills-based distress reduction in oncology) based on the psycho-oncological content topics identified in step 1. The Make It Training and the data of its first pilot study were described in depth by Ringwald et al. (48). The first version of the interactive, web-based Make It Training included eight modules and different media such as tutorial videos, audios, and individual exercises for training skills regarding the presented psycho-oncological content topics. The eight modules were designed to be completed over 4 months.

The first study assessing user acceptance showed considerable acceptance and satisfaction by cancer-affected patients (48). The training helped patients deal with disease-related burdens, and more than half of the respondents expressed the desire to attend the Make It Training further. Over 77% of the respondents would recommend the Make It Training to other patients and think that the training would add significant value to the current psycho-oncological care. However, additional contents were desired and recommended by the patients. Subsequently, we conducted a longitudinal observational study to evaluate the Make it Training’s usability, feasibility, and sustainability (45). The overall aim of these two studies was to enhance and further develop the Make It Training based on the patients’ outcomes and personal feedback.

### Step 3—Results of the patient council involvement

The task of the patient council was to accompany the study through regular meetings and communication with the study team. They advised the research team regarding (1) the optimization of the digital training and the study process, (2) a patient-friendly user experience, and (3) the dissemination of the project among patients and self-help groups. The council members were previously or still are affected by a cancer disease and are interested in science and supporting psycho-oncological care. The process of patient involvement was in line with the Involve guidelines issued by a work group of the British National Institute for Health Research (49).

Constant exchange and meetings with the patient council and the research team have occurred. Due to the COVID-19 pandemic, these meetings were held online. An overview of the cooperation process is presented in Table 1.

**TABLE 1:** Overview of the cooperation with the patient council.

Topics	Participants	Type of communication
General background information regarding online interventions and gamification tools	Patient council, research team, IT and informatics team	Online meeting
Desirable features for the intervention	Group discussion: Patient council, research team, IT and informatics team	Online meeting
Contents and design of the intervention	Patient council, research team, IT and informatics team	Online meetings with the group and single members, constant exchange *via* phone calls, e-mail exchange
Usability and design of the web app	Patient council, research team, IT and informatics team	Online meeting with the group and single members, phone calls, e-mail exchange
Design and contents of the study website	Patient council, research team	Online meeting
Effective promotion and publicity strategies	Patient council, research team	Online meetings with single members, phone calls, e-mail exchange
Questionnaires for evaluation	Patient council, research team	Online meeting with the council, phone calls, e-mail exchange

### Step 4—Content development of the make it training optimized

**FIGURE 2:**
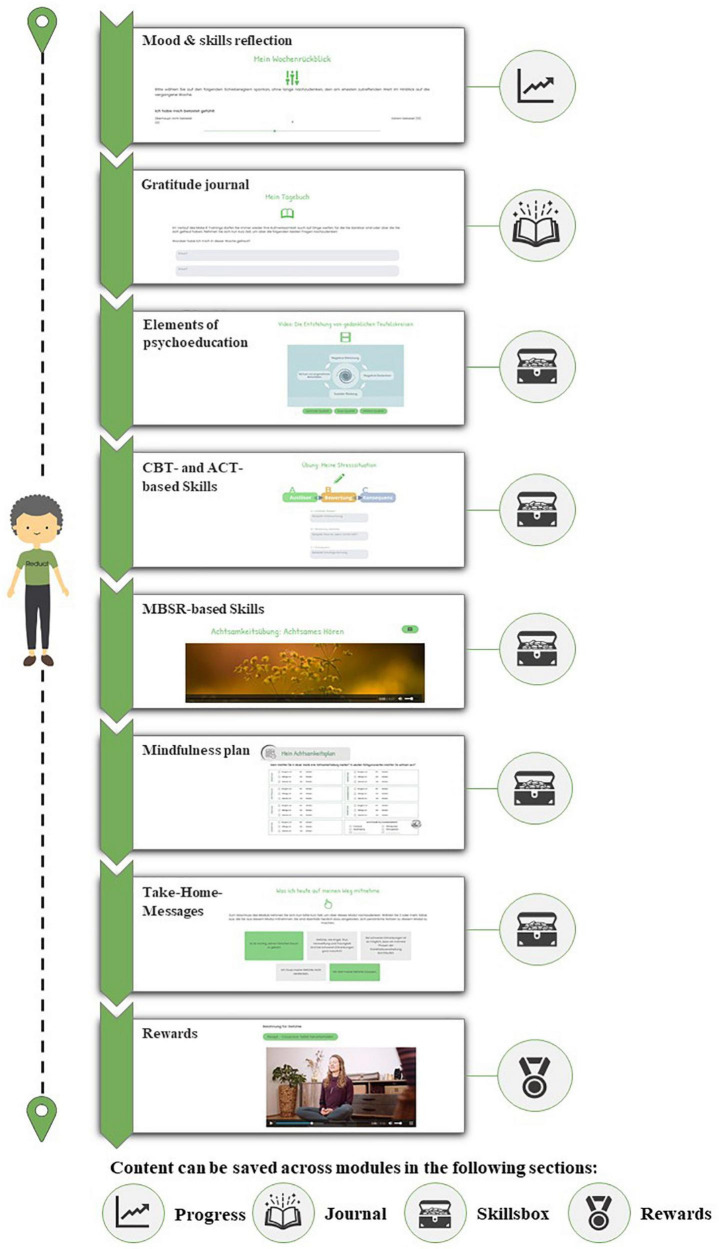
Structure of the Make It Training.

In the following, the intervention is described according to the TIDieR checklist [(46), see Supplementary Table 1]. Based on steps 1–3, the Make it Training was further developed into a self-guided and interactive digital intervention. In addition to the eight original modules (mandatory modules), it was complemented by eight optional add-on modules, making it a total of 16 modules (Supplementary Table 2 provides an overview of the modules). Patients are encouraged to complete all of the mandatory modules. The optional modules can be chosen according to personal needs to allow for personalization, thereby enhancing the personal relevance of the contents. The content of the modules was adapted to the demands and needs that we surveyed and evaluated (see steps 1–3). The aim of all modules is that patients learn how to cope with their fears and worries during the cancer trajectory and to improve their wellbeing and quality of life. Each module of the Make it Training optimized is structured identically (see Figure 2). It consists of psychoeducation, skills training, and mindfulness. The module starts with a short self-evaluation of current distress level, skills, and mindfulness, so the patient can reflect on their process of disease coping. The progress concerning these three measures is visualized in a graph. In the second step, patients are asked to answer two questions in a gratitude journal. This way, they are encouraged to reflect on the positive aspects and events of the last week. Each module contains psychoeducational videos, skills training, audio-guided mindfulness exercises, and an individualized summary of the completed module. The psychoeducational videos consist of custom animated clips and interviews with healthcare professionals. The animated clips have been created using the PowToon Software (50). The interviews with the healthcare professionals were recorded using the DJI Osmo Pocket camera and edited using the iMovie Software (51). The skills training involves exercises that help patients acquire strategies to support disease-related coping. Some exercises can be downloaded as a PDF to encourage repetition throughout the week and beyond completing the Make It Training optimized. Supplementary Table 2 shows which exercises and therapeutic methods have been used in the modules. The patients can store helpful information, materials, and strategies in an individualized skills box. This skills box allows the patients to collect a personally relevant set of knowledge and skills they can fall back on beyond completion of the training. Patients can access the skills box at any time. At the end of each module, the patient receives a mindfulness exercise plan and motivational messages to encourage the practice of formal and informal mindfulness throughout the week. These materials can be downloaded and used offline. The Make It Training optimized supports the patients over the course of 4 months. Each week a new module is unlocked to accompany patients on their way through the cancer disease in the coming next week. The completion of a module takes around 20–30 min (for screenshots of the described intervention elements, please consult the Supplementary material).

### Step 5—Technical implementation of the web-based intervention

The implementation of the intervention into a web app was realized by a team of health informatics scientists and graphic designers of the Fraunhofer Institute for Software and System Engineering (ISST). The Fraunhofer ISST specializes in the research and development of software technology for digital and data-driven healthcare. Furthermore, the patient council was regularly asked for feedback following new development steps or updates on the intervention. Overall, it took around 4 months to implement the intervention. The web framework *Vaadin Version 14* (52) was used to develop the web app’s front and back end. The NoSQL Database *MongoDB 5.0* (53) was used for authorization management and progress tracking within the intervention. In this implementation phase, weekly interdisciplinary meetings took place. During those meetings, relevant aspects of the user experience, graphic design, and other feedback were reviewed. Especially the feedback intervals between the healthcare specialists, informatics specialists, and patient council helped improve the intervention further and adapt it to cancer patients’ needs.

The intervention was developed as a web app, as this format allows the use of it on different electronic devices such as laptops, computers, mobile phones, or tablets. The web app can be accessed *via* the study website.^1^ The study website is the central contact point for interested patients and healthcare professionals. Information about the ongoing effectiveness trial Reduct (45), the research team, cooperation partners, terms of participation, and media presence can be accessed there. To register for the Make It Training optimized, patients receive an access code from the study team *via* e-Mail to create an account. Data safety is ensured by pseudonymizing patient data, meaning that gathered data cannot be traced back to the individual participants.

The Make It Training optimized can be used *via* different web browsers—Google Chrome, Safari, and Firefox. To ensure the quality of the web app, IT specialists, the research team, and members of the patient council independently tested the intervention’s functionality and usability throughout the development process. Regular exchanges took place to test updated features and optimize the intervention.

### Step 6—Motivation and evaluation plan

To ensure that the Make It Training optimized is intuitively usable, the process and structure of the modules are always the same. Each module is designed to be completed over a week, and a new module is activated weekly. To promote adherence, the users receive a standardized reminder e-mail with a motivating mindful quote and a short teaser about the module’s content when a new module is unlocked. After 2 and 4 weeks of inactivity, users receive e-mails after 2 and 4 weeks to remind them of the available module and motivate them to participate. In addition, we have incorporated elements of gamification, which have been linked to enhanced motivation (54). At the beginning of the Make It Training optimized, the users can choose their personalized avatar to accompany them on their journey of coping with the disease throughout the modules. During the modules, the avatar provides standardized positive feedback and advice and draws attention to important information. The avatar acts as a friendly companion and gives positive feedback when a module is completed. After completing the module, the avatar moves on to the next module, so the users can see their progress in the Make It Training optimized (Figure 2 shows the gamification process and the avatar). After completing a module, the patients receive two rewards: a cooking recipe and a yoga video. The yoga videos were recorded and made available to us by a certified yoga teacher. The recipes were developed by a cook and nutritional expert together with an oncologist.

To evaluate the intervention, we are conducting a randomized controlled trial. The intervention group receives the Make it Training optimized. In contrast, the control group only receives brief information and tips for coping with illness (further descriptions can be found in the study protocol, 46). To assess the efficacy and usability of the intervention, we used patient-reported outcomes measures (PROMs) and patient-reported experience measures (PREMs) to evaluate the web app, which is described in-depth in the published study protocol (45).

## Discussion

Cancer-affected patients often suffer from high distress (1, 2). So far, there is a research gap concerning the evaluation of web-based psycho-oncological support. This protocol provides a detailed account of the different steps of developing an innovative, web-based intervention to fill this research gap.

In six steps, we developed an evidence-based, patient-oriented intervention, the Make It Training optimized. In the first step, the needs and demands assessment revealed relevant psycho-oncological content topics and showed demand for eHealth applications in psycho-oncological care. The first version of the interactive, web-based Make It Training was developed in a second step. A first acceptance analysis showed high acceptance and provided feedback for further development. The third step was the implementation and involvement of the patient advisory council, which contributed to the further development and evaluation of the web-based intervention. Step 4 constituted the further refinement of the Make It Training, resulting in the Make It Training optimized. The result was a multifaceted training that addresses 16 topics relevant to cancer patients. In step 5, we implemented the web app of the intervention and the study website. And last, step 6 led to gamification and notification to motivate patients and enhance their adherence.

### Strengths and limitations

To develop a web-based psychotherapeutic intervention, it is essential to involve cancer-affected patients from the beginning. In doing so, we created a web-based intervention that addresses the demands and needs of cancer-affected patients. Due to the constant exchange and feedback processes with the patient council, user difficulties were identified, and constructive solutions were found in a cooperative exchange. In addition, the dissemination of the intervention was improved, and it was possible to draw the attention of cancer-affected individuals to the intervention. Furthermore, successful cooperation in an interdisciplinary team with different expertise, i.e., psycho-oncologists, psychotherapists, medical specialists, graphic designers, and health informatics scientists, was central to developing an effective web-based psycho-oncological intervention. Only through this interdisciplinary cooperation was it possible to ensure that patients could optimally benefit from the intervention in the context of their disease management.

Our intervention constitutes a self-guided intervention without therapist contact. It is designed to help cancer patients manage disease-related challenges autonomously. While not all cancer patients need or wish for in person psychological support, it cannot replace face-to-face interventions altogether. This especially applies to patients suffering from severe mental disorders or impairments who require more extensive support. In addition, while we strived to make the intervention as intuitively usable as possible, the use of intervention calls for a certain affinity with using web-based applications. One challenge of developing e-mental health interventions is to compete with existing commercial products in terms of available resources. This might influence the web design and usability of the intervention compared to commercial products in the e-mental health sector. Furthermore, critical readers might point out that combining different psychotherapeutic methods complicates identifying which therapeutic elements of the intervention were helpful. However, the three therapeutic techniques have a large evidence base for psychological support of cancer patients (27, 34, 37, 41, 42). By combining them and introducing a personal skills box, we made sure that patients can explore different methods to deal with their illness and personally select what helps them best. A general difficulty posed by e-health products is their standardized nature. Making one size fit all is impossible, and experts highlight the need for tailoring e-mental health interventions to individual needs (13, 55, 56).

## Conclusion and implications for future research

With the help of this protocol, researchers and the general public can gain further insights into the process and structure of our web-based psych-oncological intervention. To develop effective e- mental health interventions, psychotherapy research must be combined with the state of the art of effective online applications to establish an evidence base for e-mental health applications. The pending results of our current randomized controlled effectiveness trial will show, whether our intervention is indeed effective and easily usable. Furthermore, qualitative research in the form of structured interviews with participants as well as moderation analyses will provide more insights into how the intervention was perceived by users. Future research should examine whether the intervention is effective for different age groups and tumor entities. Furthermore, dismantling studies could shed further light on the potential of this multimodal approach for cancer patients. Lastly, tools to further personalize e-mental health interventions, such as individually tailored feedback and content (13, 57, 58), should be explored to increase adherence and effectiveness. At the same time, the publication of intervention protocols could increase transparency and help researchers in the same field better understand what works and does not. Ultimately, these research efforts will inform the design of effective web-based psycho-oncological interventions to complement existing face-to-face support.

## Data availability statement

The original contributions presented in the study are included in the article/Supplementary material, further inquiries can be directed to the corresponding author/s.

## Ethics statement

The studies involving human participants were reviewed and approved by the Medical Faculty, University Hospital Tübingen, Eberhard Karls University; Medical Faculty, University of Duisburg-Essen, Essen, Germany; Medical Faculty, University Hospital Erlangen, Friedrich-Alexander-University Erlangen-Nürnberg; Medical Faculty, University Medical Center Leipzig, Leipzig, Germany; Medical Faculty, University Medical Center of the Johannes Gutenberg-University Mainz, Mainz, Germany; Medical Faculty, Albert-Ludwigs-Universität Freiburg, Freiburg Medical Center, Freiburg, Germany; Medical Faculty, Klinikum Rechts der Isar, School of Medicine, Technical University of Munich, München, Germany; Medical Faculty, Justus Liebig University Giessen, Giessen, Germany; and Medical Faculty, Philipps University Marburg, Marburg, Germany. The patients/participants provided their written informed consent to participate in this study.

## Author contributions

MT, AB, JG, and YE obtained the funding. SZ was a senior consultant within the project and provided supervision. JG, JH, AB, AW, CS, JK, JB, SS, and CJ developed and further adapted the intervention and prepared the content for the e-health application. SD and MP were responsible for implementing the intervention into a web app. JG and JH drafted the manuscript. AB, JK, and CS drafted the sections specifying the app development and patient council involvement. AM-T, JW, AW, AD, AS, and JK oversaw the recruitment at their study site and provided feedback during intervention development and contributed to this manuscript. All authors critically reviewed the final version of the manuscript and approved it and contributed to the article and approved the submitted version.

## Footnotes

1
https://app.reduct-studie.de/login
