# Supplementary material for: Mindfulness and skills-based eHealth intervention to reduce distress in cancer-affected patients in the Reduct trial: Intervention protocol of the make it training optimized
Source: Front Psychiatry. 2022 Oct 28;13:1037158. doi: 10.3389/fpsyt.2022.1037158 (PMC9650647; doi:10.3389/fpsyt.2022.1037158)
Supplement: Supplementary file 1 [file Data_Sheet_1.pdf]

Supplementary Material

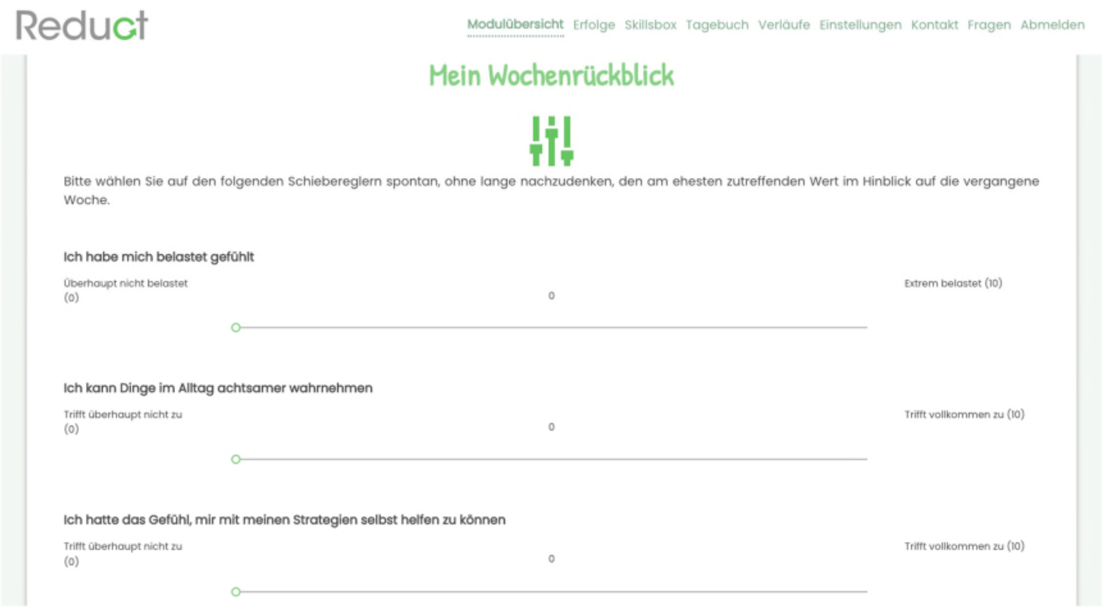

Supplementary Screenshot 1. Self-evaluation of Distress Level, Skills and Mindfulness

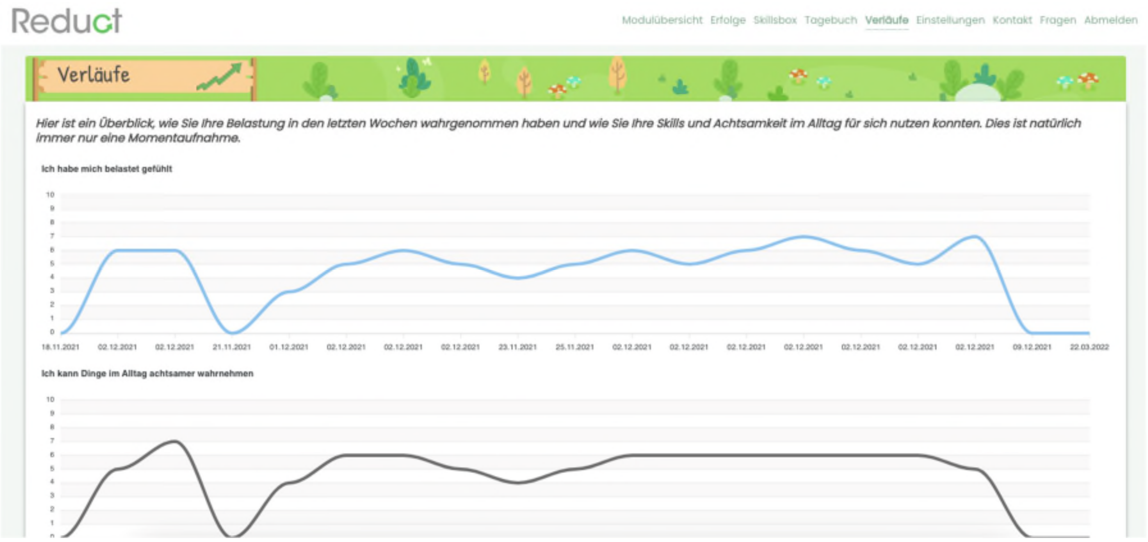

Supplementary Screenshot 2. Visualization of Progress

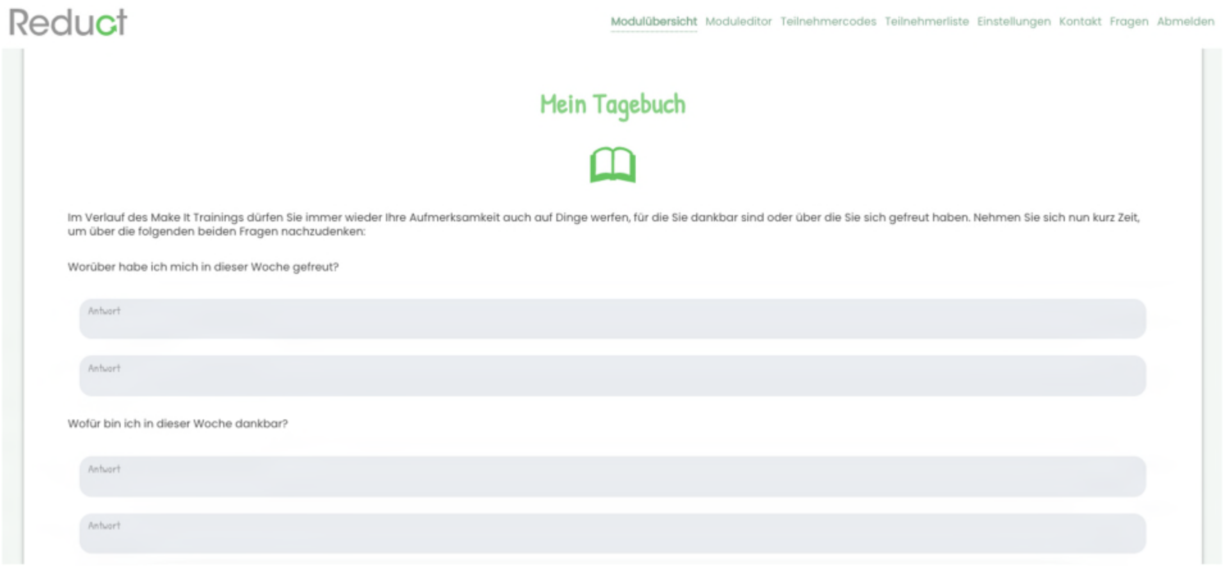

Supplementary Screenshot 3. Gratitude Journal

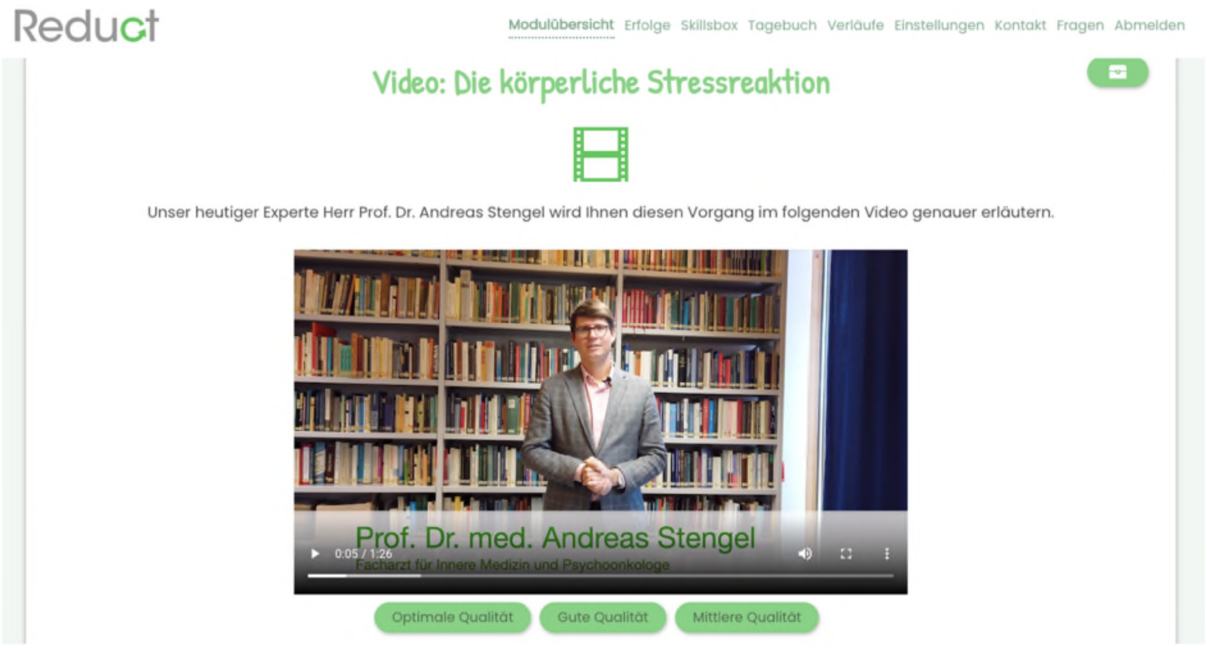

Supplementary Screenshot 4. Psychoeducational Video With a Healthcare Professional

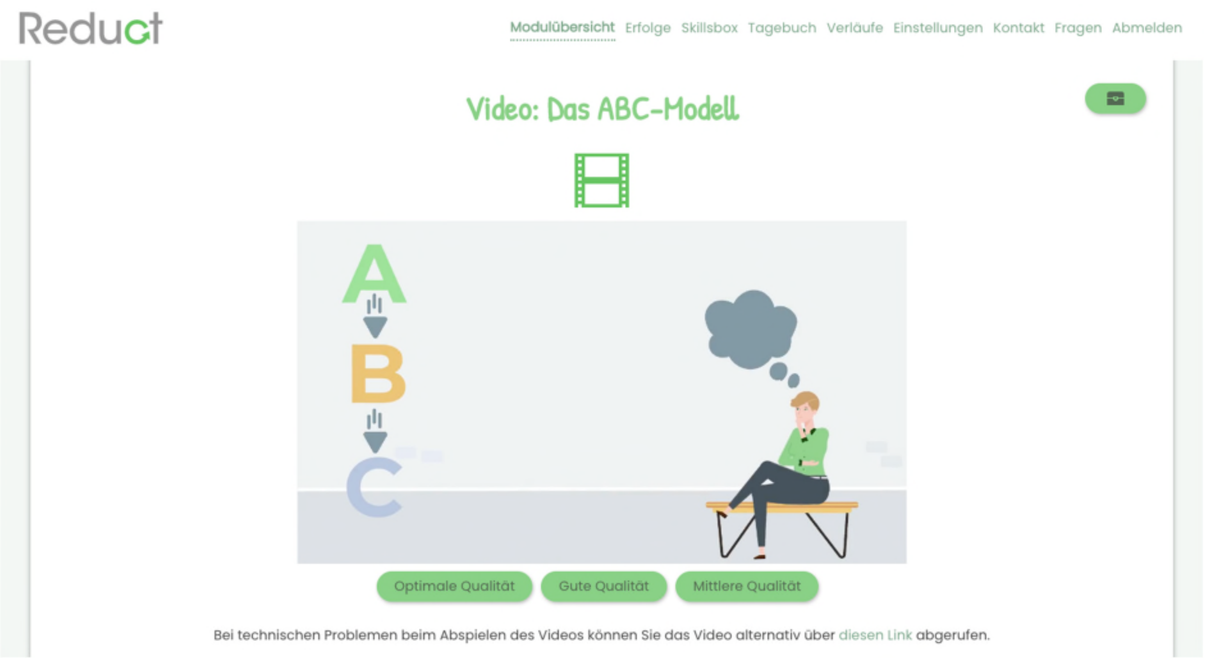

Supplementary Screenshot 5. Psychoeducational Video: Custom Animated Clip

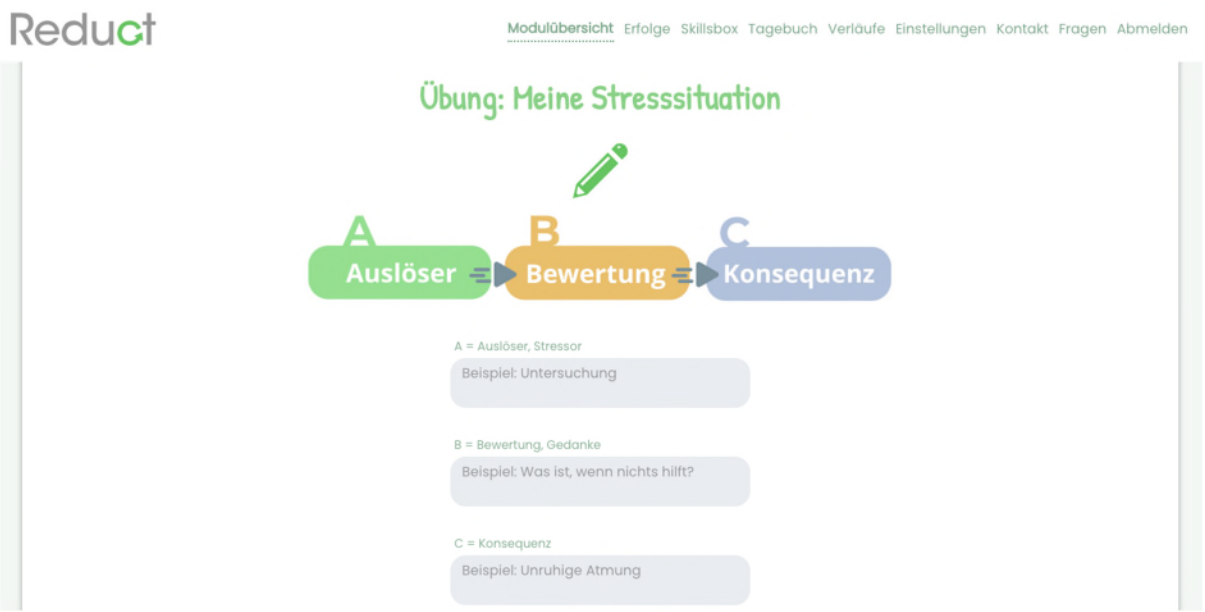

Supplementary Screenshot 6. Skills Training

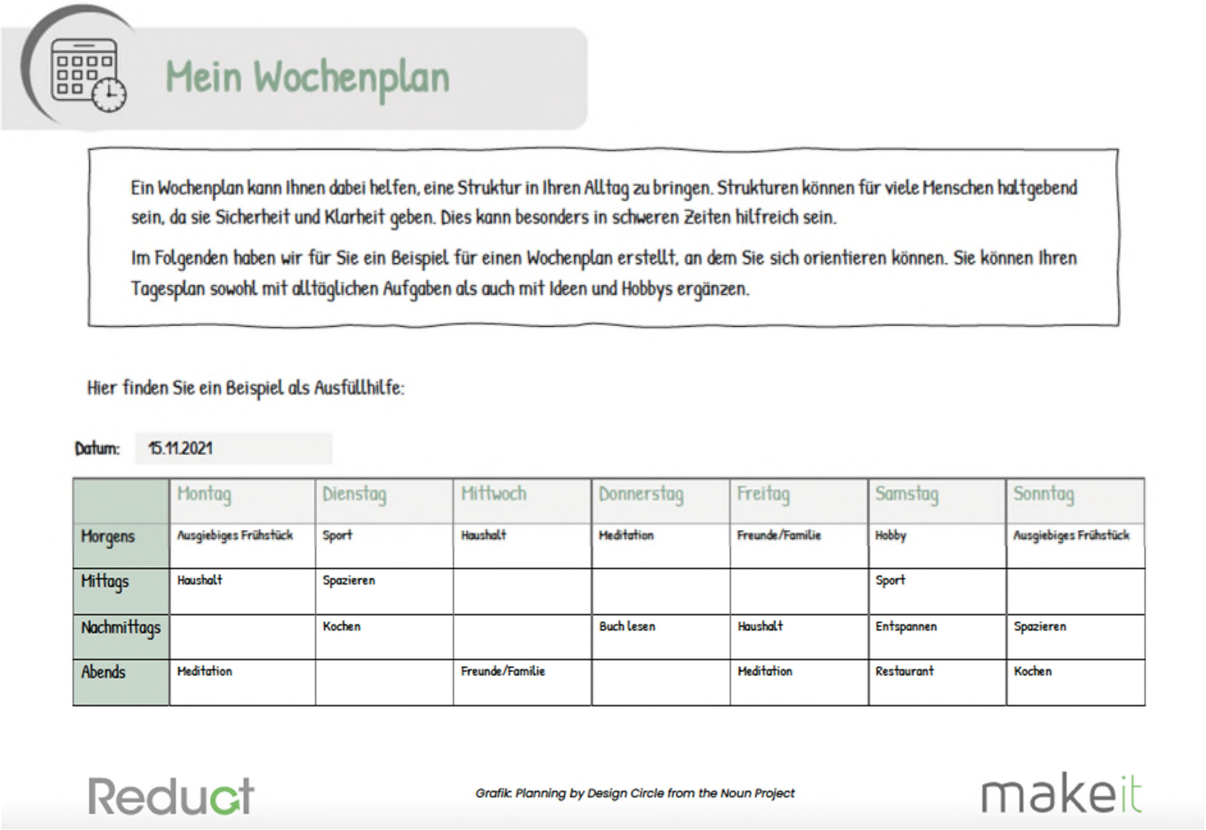

Mein Wochenplan

Ein Wochenplan kann Ihnen dabei helfen, eine Struktur in Ihren Alltag zu bringen. Strukturen können für viele Menschen haltgebend sein, da sie Sicherheit und Klarheit geben. Dies kann besonders in schweren Zeiten hilfreich sein.

Im Folgenden haben wir für Sie ein Beispiel für einen Wochenplan erstellt, an dem Sie sich orientieren können. Sie können Ihren Tagesplan sowohl mit alltäglichen Aufgaben als auch mit Ideen und Hobbys ergänzen.

Hier finden Sie ein Beispiel als Ausfüllhilfe:

Datum: 15.11.2021

	Montag	Dienstag	Mittwoch	Donnerstag	Freitag	Samstag	Sonntag
Morgens	Ausgiebiges Frühstück	Sport	Haushalt	Meditation	Freunde/Familie	Hobby	Ausgiebiges Frühstück
Mittags	Haushalt	Spazieren				Sport	
Nachmittags		Kochen		Buch lesen	Haushalt	Entspannen	Spazieren
Abends	Meditation		Freunde/Familie		Meditation	Restaurant	Kochen

Grafik: Planning by Design Circle from the Noun Project

Supplementary Screenshot 7. Downloadable Exercises

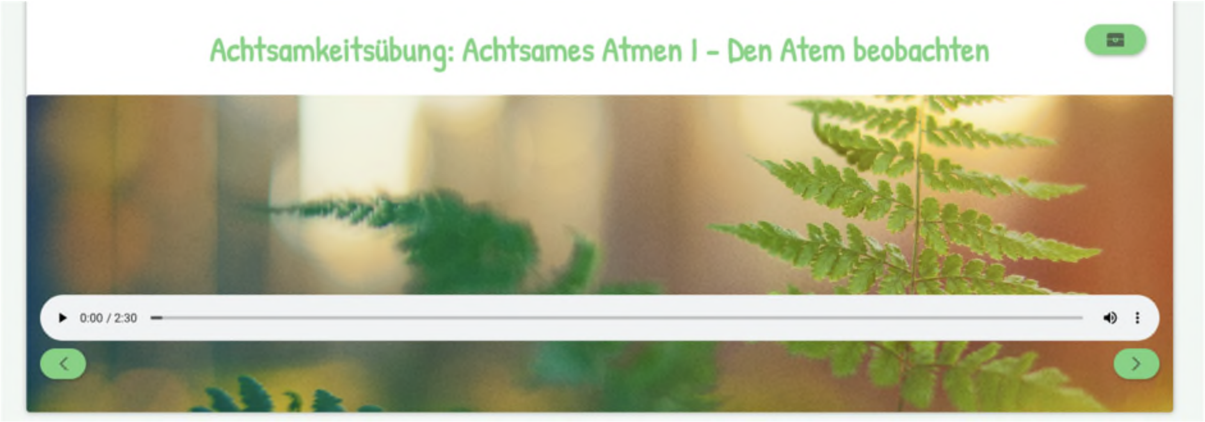

Supplementary Screenshot 8. Audio-guided Mindfulness Exercises

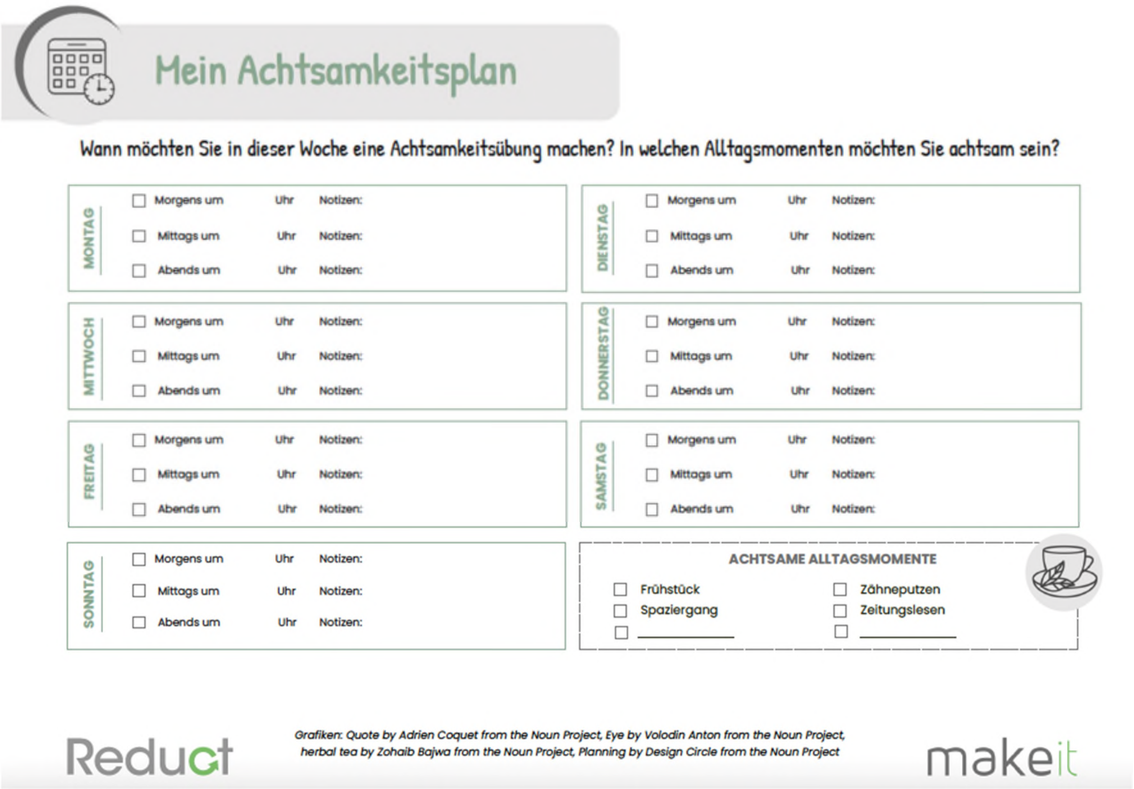

Supplementary Screenshot 9. Mindfulness Exercise Plan

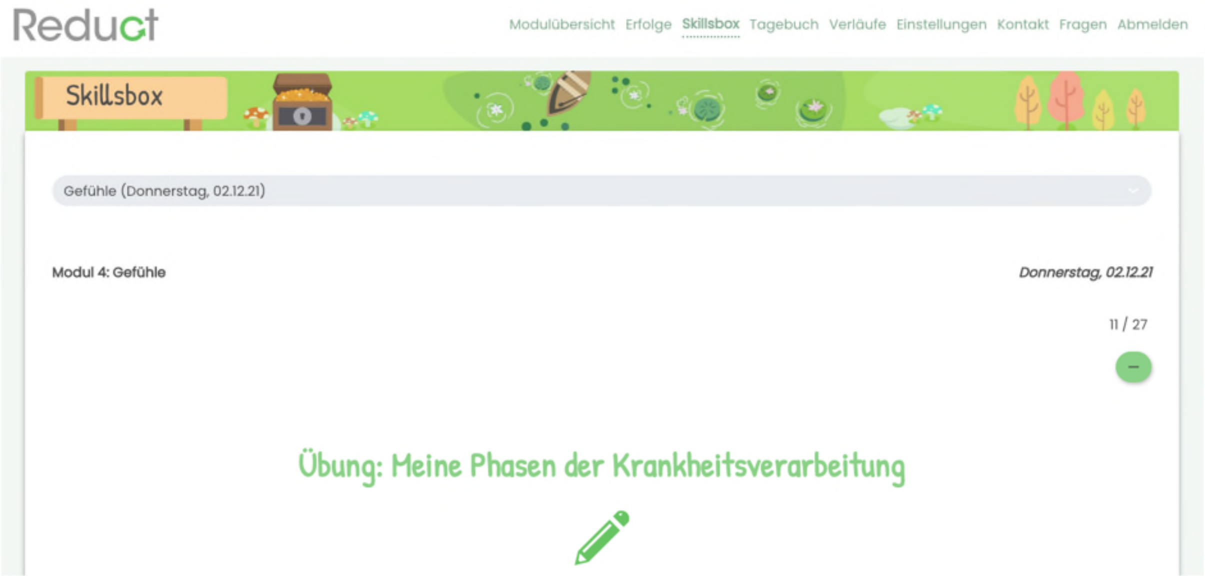

Supplementary Screenshot 10. Skillsbox

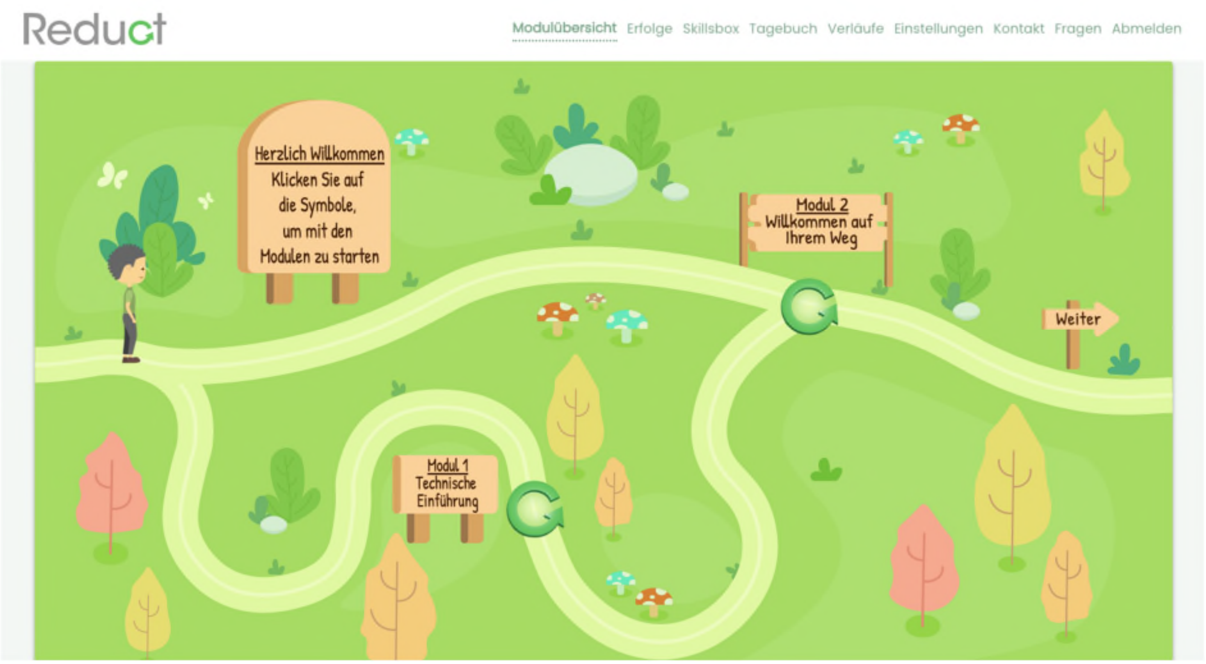

Supplementary Screenshot 11. Avatar 1

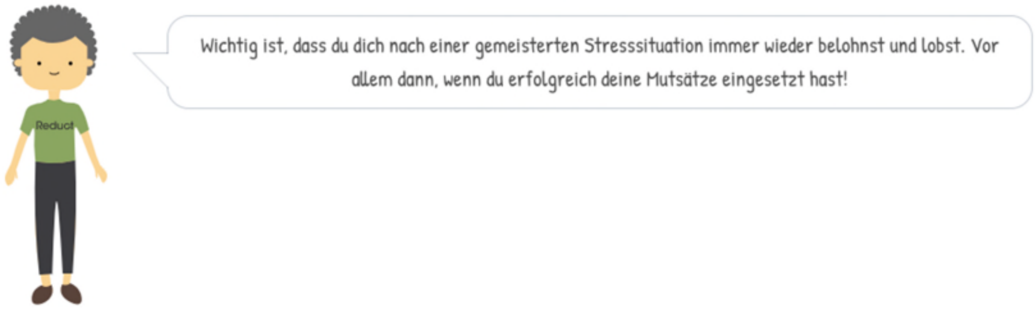

Supplementary Screenshot 12. Avatar 2

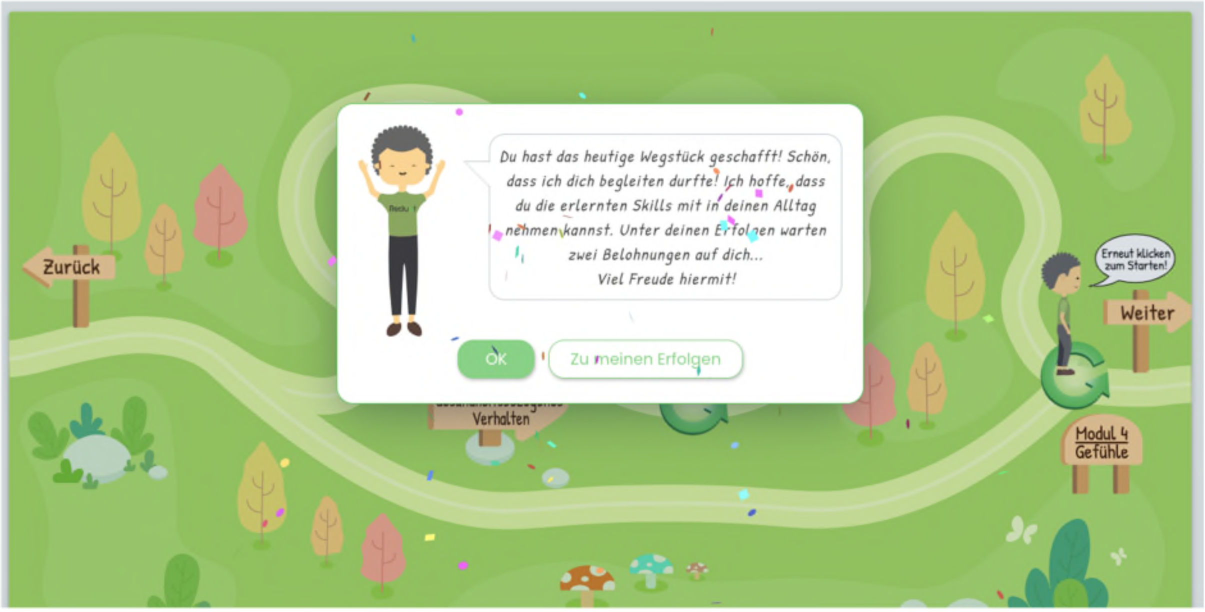

Supplementary Screenshot 13. Rewards 1

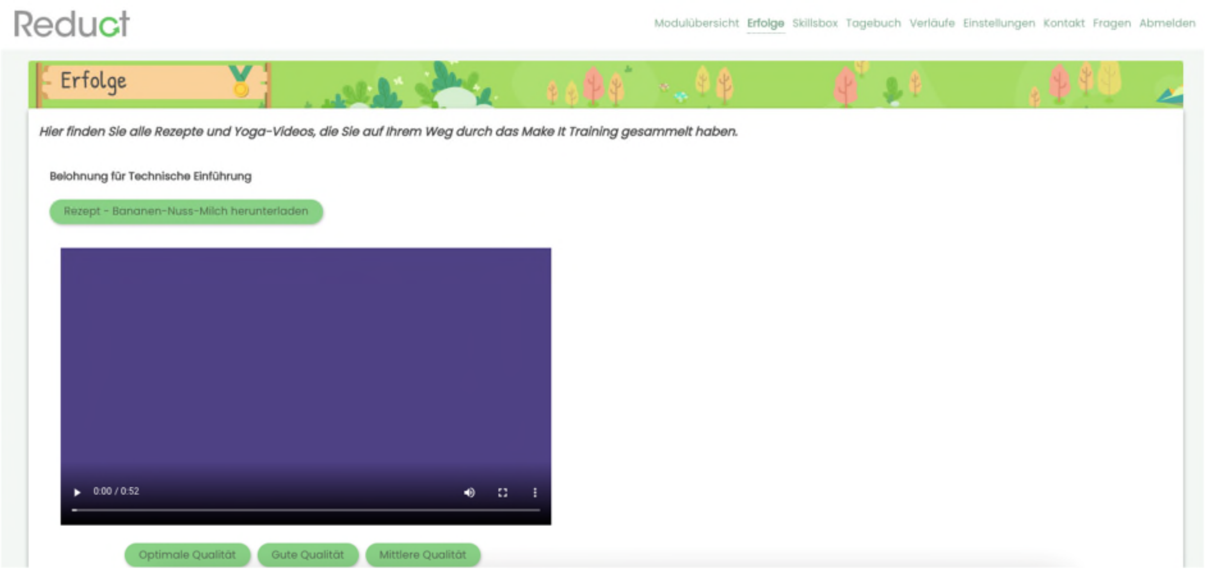

Supplementary Screenshot 14. Rewards 2
