# Supplementary material for: Mindfulness and skills-based eHealth intervention to reduce distress in cancer-affected patients in the Reduct trial: Intervention protocol of the make it training optimized
Source: Front Psychiatry. 2022 Oct 28;13:1037158. doi: 10.3389/fpsyt.2022.1037158 (PMC9650647; doi:10.3389/fpsyt.2022.1037158)
Supplement: Supplementary file 3 [file Table_2.pdf]

frontiers

Supplementary Material

Supplementary Table 2. Overview of the module contents

	Module	Topic	Skills			Mindfulness	
			Psychoeducation	Video(s)	Skills-Exercise(s)	Topic	Audio-guided Practice
1	Technical Introduction	A quick introduction to the app	/	Technical Introduction Video (How to use the Make It Training)[2]	/	/	/
**2**	**Welcome to your Path**	**Introduction to the structure of the Make It Training and to Mindfulness**	**Introducing:**<br>**- The Make It Training: What can participants expect**<br>**- The gratitude Journal**<br>**- Mindfulness practice and its benefits**	**1. Introducing the Make It Training (Prof. Dr. Martin Teufel, Dr. Alexander Bäuerle)[1]**<br>**2. Introducing Margit K.[1]**<br>**3. What is mindfulness?[2]**<br>**4. Practicing Mindfulness[2]**	**1: My goal(s) for the Make It Training**	**Observing the Breath to anchor in the moment**	**Body Scan II – Mindful Body Awareness**
3	Health-related Behavior	Different aspects of promoting physical and mental health during cancer illness	- creating a daily structure<br>- a healthy diet<br>- dealing with online health information	1: A healthy diet in the context of cancer (Kristine Schweins)[1]	1: Planning my day	Anchoring in the moment by paying attention to our senses non-judgmentally - smelling	Mindful Smelling
**4**	**Emotions**	**Accepting and dealing with emotions as a part of processing the cancer illness.**	**- The four stages of dealing with cancer**<br>**- Making room for emotions**	**1: The four stages of dealing with cancer (Prof. Dr. Yesim Erim)[1]**<br>**2: The four stages of dealing with cancer (Summary)[2]**	**Exercise 1: My phases of dealing with cancer**<br>**Exercise 2: The Room of emotions**	**Mindfully experiencing thoughts and emotions to distance from them**	**Mindful Experiencing**

	Module	Topic	Skills			Mindfulness	
			Psychoeducation	Video(s)	Skills-Exercise(s)	Topic	Audio-guided Practice
5	Fear	Dealing with cancer-related fears including fear of progression.	- Why we experience Fear<br>- Fear of Progression	1: Why we experience fear and the role of our thoughts[2]<br>2: Meeting todays expert: Dr. Monika Schmid[1]<br>3: Coping with fear of progression (Dr. Monika Schmid)[1]<br>4: Using Mindfulness to cope with fear (Dr. Monika Schmid)[1]	1: My experience of fear of Progression<br>2: Where do I feel fear? (And when)<br>3: My emergency case	Using a visualization to take the weight from distressing thoughts and emotions	Thoughts to Feathers
**6**	**Pain**	**Understanding and dealing with cancer-illness- and treatment-related pain.**	**- Pain and cancer**<br>**- The function of pain**<br>**- Coping strategies**<br>**• Thought stopping**<br>**• Guiding attention**	**1: How pain emerges (Dr. Caroline Rometsch)[1]**<br>**2: Acute vs recurring pain[2]**<br>**3. Coping with pain (Dr. Caroline Rometsch)[1]**<br>**4. Thought stopping[2]**	**1: Pain Journal**<br>**2: Thought-stopping technique**	**Observing bodily sensations non-judgmentally**	**Body Scan I- Mindful Body experience**
7	Sleep	Foundations of restful sleep: Strategies and the role of thoughts and emotions that arise	- How cancer can affect sleep<br>- What makes sleep restful<br>- Strategies for restful sleep<br>• Thought stopping<br>• Creating a designated place for worrying<br>• Adopting an evening routine to set the stage for restful sleep	1: How thoughts can impact our sleep[2]<br>2: Thought stopping (repetition)[2]<br>3: Creating a place for worrying[2]<br>4: More Tipps for falling and staying asleep (Dr. Christoph Schöbel)[1]	1: Collection of distressing thoughts<br>2: Thought stopping (repetition)<br>3: Bedtime routine	Using a visualization to let go of thoughts and emotions	Mindfully Letting Go
**8**	**Activating Resources**	**Discovering and consciously using personal resources of strength to cope with cancer**	**The vicious circle of thoughts**	**1: The circle of thoughts[2]**<br>**2: Using personal resources (Martina Madl)[1]**<br>**3: Quitting the thought circle[2]**	**1: My Circle of Thoughts**<br>**2: Quitting the thought circle**<br>**- Seeking consolation/support**<br>**- Accepting emotions**<br>**- Planning social activities**	**Anchoring in the moment by paying attention to our senses non-judgmentally - *hearing***	**Mindful hearing**

					**- Planning joyful activities**		
			**Skills**			**Mindfulness**	
	**Module**	**Topic**	**Psychoeducation**	**Video(s)**	**Skills-Exercise(s)**	**Topic**	**Audio-guided Practice**
9	Positivity	Making room for positive experiences; noticing accomplishments and step-by-step planning.	- Room for positivity<br>- What positivity means (and what it doesn´t mean)	1: Step by Step[2]	1: Which steps have I mastered? (Reflecting on successes)<br>2: Focusing on the next step<br>3: Using affirmative sentences	Becoming aware and fully experiencing beautiful moments	Mindful Moment
**10**	**Body Awareness**	**Dealing with cancer-related bodily changes**	**- How cancer can affect the body (and body perception)**<br>**- Dealing with bodily changes**	**1: Body Perception[2]**<br>**2: Dealing with bodily changes (Dr. Kaya)[1]**	**1: How did my body change?**<br>**2: The weighing pan**<br>**3: Bodily changes as a part of my cancer journey**<br>**- What me and my body have accomplished**<br>**- Taking care of my body**<br>**- Paying attention to body signals**	**Noticing sensations and needs of my body non-judgmentally**	**Body Scan II – Mindful Body Awareness**
11	Exercise and Relaxation	Finding a healthy balance between exercise and relaxation as part of recovery from cancer.	- Exercise in the context of cancer: Benefits and things to consider<br>- Balancing exercise and relaxation		1: Small changes with big impact (Incorporating brief elements of exercise into everyday life)<br>2: My energy balance<br>- Energy takers<br>- Energy gainers	Using the power of the Breath to relax	Mindful Breathing II – Triangle Breath
**12**	**Stress Management**	**Mastering stressful cancer-related situations**	**- The significance of thoughts for subjective experience and feelings of distress (ABC Model)**<br>**- Managing stress based on affirmative thoughts**	**1: The physical stress reaction (Prof. Dr. Andreas Stengel)[1]**<br>**2: The ABC Model of Experiencing stress[2]**<br>**3: Dealing with stress[2]**	**1: My Stress situation (ABC Model)**<br>**2: Using alternative/affirmative thoughts**	**Anchoring in the moment by paying attention to our senses non-judgmentally - *vision***	**Mindful Vision**

	Module	Topic	Skills			Mindfulness	
			Psychoeducation	Video(s)	Skills-Exercise(s)	Topic	Audio-guided Practice
13	Creativity	Exploring creativity as a resource of strength during cancer illness.	- Introducing Art Therapy and its benefits	1: Insights into art therapy (Edith Stosiek)[1]<br>2: Instruction: Mindful Doodling (Edith Stosiek)[1]	1: When was the last time I engaged in creativity?	Using the power of doodling without focusing on the result to anchor in the moment and relax	Mindful Doodling
**14**	**Self-care**	**Self-appreciation and self-care and their importance in the context of cancer**	- **The pillars of self-esteem.**	**1: The pillars of self-esteem**[2]<br>**2: The foundations of self-esteem (Dr. Johanna Graf)**[1]	**1: My pillars of self-esteem**<br>**2: Strengthening our foundation of self-esteem**<br>- **Self-care**<br>- **Self-Love/Kindness**<br>- **Mindfulness**	**Fostering self-compassion through mindfulness**	**Mindful Self-Compassion**
15	Relationships	Personal relationships as a resource and communication of health information and need toward family and friends in the context of the cancer illness	1. The Partner<br>2. Children<br>3. Peers/Social Network	1: Introducing todays expert: Family and Couples therapist Mette Barslev[1]<br>2: The relationship to our partner (Mette Barslev)[1]<br>3: How the cancer illness of a parent can impact children (Mette Barslev)[1]<br>4: How can I lean on my peers? (Mette Barslev)[1]	1: Partner Exercise (Communicating needs and boundaries, exploring shared values and resources)<br>2: Communicating with peers	Fostering the connection to oneself and others with loving kindness	Loving-Kindness
**16**	**Looking back**	**Review of the Make It Training and looking ahead**	- **Reflecting on helpful skills**<br>- **Looking ahead: Using skills beyond the training (Skillsbox)**	**1: Using the skills box beyond the Make It Training (Prof Dr. Stephan Zipfel)**[1]<br>**2: Practicing Mindfulness on the road ahead (Dr. Johanna Graf)**[1]	**Choosing Take Home messages**	**Choosing a favorite exercise as part of the reflection**<br>**OR**<br>**BONUS exercise: Mindful walk**	**Favorite Mindfulness Practice OR Mindful Walk**

Note. **Mandatory Module,** Optional Module; [1] Camera recorded expert Videos, [2] explanatory cartoon videos created with the software *Powtoon*
