# Supplementary material for: Mindfulness and skills-based eHealth intervention to reduce distress in cancer-affected patients in the Reduct trial: Intervention protocol of the make it training optimized
Source: Front Psychiatry. 2022 Oct 28;13:1037158. doi: 10.3389/fpsyt.2022.1037158 (PMC9650647; doi:10.3389/fpsyt.2022.1037158)
Supplement: Supplementary file 4 [file Table_3.docx]

Supplementary Material

**Supplementary Table 3.** Overview of the module contents

|  | **Module** | **Topic** | **Skills** | | | **Mindfulness** | |
| --- | --- | --- | --- | --- | --- | --- | --- |
|  |  |  | **Psychoeducation** | **Video(s)** | **Skills-Exercise(s)** | **Topic** | **Audio-guided Practice** |
| 1 | Technical Introduction | A quick introduction to the app | / | Technical Introduction Video (How to use the Make It Training) | / | / | / |
| **2** | **Welcome to your Path** | **Introduction to the structure of the Make It Training and to Mindfulness** | **Introducing:**   - **The Make It Training: What can participants expect** - **The gratitude Journal** - **Mindfulness practice and its benefits** | 1. **Introducing the Make It Training^1^** 2. **Introducing Margit K.^1^** 3. **What is mindfulness?^2^** 4. **Practicing Mindfulness^2^** | **1: My goal(s) for the Make It Training** | **Observing the Breath to anchor in the moment** | **Body Scan II – Mindful Body Awareness** |
| 3 | Health-related Behavior | Different aspects of promoting physical and mental health during cancer illness | - creating a daily structure - a healthy diet - dealing with online health information | 1: A healthy diet in the context of cancer | 1: Planning my day | Anchoring in the moment by paying attention to our senses non-judgmentally - smelling | Mindful Smelling |
| **4** | **Emotions** | **Accepting and dealing with emotions as a part of processing the cancer illness.** | - **The four stages of dealing with cancer** - **Making room for emotions** | **1: The four stages of dealing with cancer (Dr. Yesim Erim)^1^**  **2: The four stages of dealing with cancer (Summary)^2^** | **Exercise 1: My phases of dealing with cancer**  **Exercise 2: The Room of emotions** | **Mindfully experiencing thoughts and emotions to distance from them** | **Mindful Experiencing** |

|  | **Module** | **Topic** | **Skills** | | | **Mindfulness** | |
| --- | --- | --- | --- | --- | --- | --- | --- |
|  |  |  | **Psychoeducation** | **Video(s)** | **Skills-Exercise(s)** | **Topic** | **Audio-guided Practice** |
| 5 | Fear | Dealing with cancer-related fears including fear of progression. | - Why we experience Fear - Fear of Progression | 1: Why we experience fear and the role of our thoughts^2^  2: Meeting todays expert: Dr. Monika Schmid^1^  3: Coping with fear of progression (Dr. Monika Schmid)^1^  4: Using Mindfulness to cope with fear ^1^ | 1: My experience of fear of Progression  2: Where do I feel fear? (And when)  3: My emergency case | Using a visualization to take the weight from distressing thoughts and emotions | Thoughts to Feathers |
| **6** | **Pain** | **Understanding and dealing with cancer-illness- and treatment-related pain.** | - **Pain and cancer** - **The function of pain** - **Coping strategies** - **Thought stopping** - **Guiding attention** | **1: How pain emerges (Dr. Caroline Rometsch)^1^**  **2: Acute vs recurring pain^2^**  **3. Coping with pain (Dr. Caroline Rometsch)^1^**  **4. Thought stopping^2^** | **1: Pain Journal**  **2: Thought-stopping technique** | **Observing bodily sensations non-judgmentally** | **Body Scan I- Mindful Body experience** |
| 7 | Sleep | Foundations of restful sleep: Strategies and the role of thoughts and emotions that arise during a | - How cancer can affect sleep - What makes sleep restful - Strategies for restful sleep - Thought stopping - Creating a designated place for worrying - Adopting an evening routine to set the stage for restful sleep | 1: How thoughts can impact our sleep^1^  2: Thought stopping (repetition)^2^  3: Creating a place for worrying^2^  4: More Tipps for falling and staying asleep (Dr. Christoph Schöbel)^1^ | 1: Collection of distressing thoughts  2: Thought stopping (repetition)  3: | Using a visualization to let go of thoughts and emotions | Mindfully Letting Go |
| **8** | **Activating Resources** | **Discovering and consciously using personal resources of strength to cope with cancer** | **The vicious circle of thoughts** | **1: The circle of thoughts^2^**  **2: Using personal resources^1^**  **3: Quitting the thought circle^2^** | **1: My Circle of Thoughts**  **2: Quitting the thought circle**   - **Seeking consolation/support** - **Accepting emotions** - **Planning social activities** - **Planning joyful activities** | **Anchoring in the moment by paying attention to our senses non-judgmentally - *hearing*** | **Mindful hearing** |
|  | **Module** | **Topic** | **Skills** | | | **Mindfulness** | |
|  |  |  | **Psychoeducation** | **Video(s)** | **Skills-Exercise(s)** | **Topic** | **Audio-guided Practice** |
| 9 | Positivity | Making room for positive experiences; noticing accomplishments and step-by-step planning. | - Room for positivity - What positivity means (and what it doesn´t mean) | 1: Step by Step | 1: Which steps have I mastered? (Reflecting on successes)  2: Focusing on the next step  3: Using affirmative sentences | Becoming aware and fully experiencing beautiful moments | Mindful Moment |
| **10** | **Body Awareness** | **Dealing with cancer-related bodily changes** | - **How cancer can affect the body (and body perception)** - **Dealing with bodily changes** | **1: Body Perception^2^**  **2: Dealing with bodily changes (Dr. Kaya)^1^** | **1: How did my body change?**  **2: The weighing pan**  **3: Bodily changes as a part of my cancer journey**   - **What me and my body have accomplished** - **Taking care of my body** - **Paying attention to body signals** | **Noticing sensations and needs of my body non-judgmentally** | **Body Scan II – Mindful Body Awareness** |
| 11 | Exercise and Relaxation | Finding a healthy balance between exercise and relaxation as part of recovery from cancer. | - Exercise in the context of cancer: Benefits and things to consider - Balancing exercise and relaxation |  | 1: Small changes with big impact (Incorporating brief elements of exercise into everyday life)  2: My energy balance   - Energy takers - Energy gainers | Using the power of the Breath to relax | Mindful Breathing II – Triangle Breath |
| **12** | **Stress Management** | **Mastering stressful cancer-related situations** | - **The significance of thoughts for subjective experience and feelings of distress (ABC Model^3^)** - **Managing stress based on affirmative thoughts** | **1: The physical stress reaction (Prof. Dr. Andreas Stengel)^1^**  **2: The ABC Model of Experiencing stress^2^**  **3: Dealing with stress^2^** | **1: My Stress situation (ABC Model)**  **2: Using alternative/affirmative thoughts** | **Anchoring in the moment by paying attention to our senses non-judgmentally - *vision*** | **Mindful Vision** |

|  | **Module** | **Topic** | **Skills** | | | **Mindfulness** | |
| --- | --- | --- | --- | --- | --- | --- | --- |
|  |  |  | **Psychoeducation** | **Video(s)** | **Skills-Exercise(s)** | **Topic** | **Audio-guided Practice** |
| 13 | Creativity | Exploring creativity as a resource of strength during cancer illness. | - Introducing Art Therapy and its benefits | 1: Insights into art therapy (Edith Stosiek)^1^  2: Instruction: Mindful Doodling | 1: When was the last time I engaged in creativity? | Using the power of doodling without focusing on the result to anchor in the moment and relax | Mindful Doodling |
| **14** | **Self-care** | **Self-appreciation and self-care and their importance in the context of cancer** | - **The pillars of self-esteem.** | **1: The pillars of self-esteem^2^**  **2: The foundations of self-esteem^1^** | **1: My pillars of self-esteem**  **2: Strengthening our foundation of self-esteem**   - **Self-care** - **Self-Love/Kindness** - **Mindfulness** | **Fostering self-compassion through mindfulness** | **Mindful Self-Compassion** |
| 15 | Relationships | Personal relationships as a resource and communication of health information and need toward family and friends in the context of the cancer illness | 1. The Partner 2. Children 3. Peers/Social Network | 1: Introducing todays expert: Family and Couples therapist Mette Barslev^1^  2: The relationship to our partner (Bette Barslev)  3: How the cancer illness of a parent can impact children  (Mette Barslev)  4: How can I lean on my peers? (Mette Barslev) | 1: Partner Exercise (Communicating needs and boundaries, exploring shared values and resources)  2: Communicating with peers | Fostering the connection to oneself and others with loving kindness | Loving-Kindness |
| **16** | **Looking back** | **Review of the Make It Training and looking ahead** | - **Reflecting on helpful skills** - **Looking ahead: Using skills beyond the training (Skillsbox)** | **1: Using the skills box beyond the Make It Training (Prof Dr. Stephan Zipfel)**  **2: Practicing Mindfulness on the road ahead (Dr. Johanna Graf)** | **Choosing Take Home messages** | **Choosing a favorite exercise as part of the reflection**  **OR**  **BONUS exercise: Mindful walk** | **Favorite Mindfulness Practice OR Mindful Walk** |

*Note.* **Mandatory Module,** Optional Module; ^1^ Camera recorded expert Videos, ^2^ explanatory cartoon videos created with the software *Powtoon*
